# Severe *Rickettsia typhi* Infections, Costa Rica

**DOI:** 10.3201/eid2911.221561

**Published:** 2023-11

**Authors:** Diana Chinchilla, Inés Sánchez, Ida Chung, Arlyn N. Gleaton, Cecilia Y. Kato

**Affiliations:** Instituto Costarricense de Investigación y Enseñanza en Nutrición y Salud, Cartago, Costa Rica (D. Chinchilla, I. Sánchez);; Centers for Disease Control and Prevention, Atlanta, Georgia, USA (I. Chung, A.N. Gleaton, C.Y. Kato)

**Keywords:** Rickettsia typhi, rickettsia, murine typhus, endemic typhus, fleaborne typhus, severe infections, bacteria, zoonoses, Costa Rica

## Abstract

Murine typhus is a febrile, fleaborne disease caused by infection with *Rickettsia typhi* bacteria. Cases can range from mild and nonspecific to fatal. We report 2 cases of murine typhus in Costa Rica, confirming the presence and circulation of *R. typhi* causing severe disease in the country.

Murine typhus is an acute febrile, fleaborne disease caused by infection with *Rickettsia typhi* bacteria. The initial symptoms are nonspecific and resemble those of other common bacterial and viral infections. Murine typhus is generally mild to moderate in severity and has a low case-fatality rate, especially in treated patients (*1**,**2*). In untreated patients, the case-fatality rate is reported to be 0.4%, and complications can occur in 6%–30% of patients (*1*).

In Costa Rica, cases of spotted fever group (SFG) rickettsiosis and Rocky Mountain spotted fever have been reported since 1950, and 6 species of *Rickettsia* have been identified (*2**,**3*). In Central America, there is serologic evidence of antibodies in humans against typhus group (TG) *Rickettsia* in El Salvador, Guatemala, Honduras, Nicaragua, and Panamá, but there are no serologic or molecular reports of *R. typhi* in Costa Rica (*2*). We report 2 cases of murine typhus in Costa Rica.

During 2015–January 2021, a total of 190 acute-phase clinical samples from patients suspected of having rickettsiosis, ehrlichiosis, Lyme disease, and other febrile illnesses were submitted to the Centro Nacional de Referencia de Bacteriología, Instituto Costarricense de Investigación y Enseñanza en Nutrición y Salud, Cartago, Costa Rica, for laboratory diagnosis. For rickettsial disease testing, we extracted DNA from whole blood, serum, swab specimens, or biopsy specimens collected <21 days from symptom onset within 3 days of collection by using the QIAamp DNA Mini Kit (QIAGEN, https://www.qiagen.com). We also used automated platforms (Seeprep12 and SGprep 32; Seegene, https://www.seegene.com) for extraction of blood and serum, according to the manufacturer’s instructions. 

We performed the *Rickettsia* genus-specific real-time PCR, PanR8, as described (*4*). We further analyzed all 8 samples that tested positive with the PanR8 assay by using the RRi6 assay; a real-time PCR assay specific for *R. rickettsii* (causative agent of Rocky Mountain spotted fever) detection (*4**,**5*). We assessed amplicon size verification by using gel electrophoresis with the Qiaxcel Automated Platform (QIAGEN). We performed immunofluorescence-IgG (Fuller Laboratories, http://www.fullerlabs.com) when second samples were available.

A total of 104 coded samples were sent to the diagnostics laboratory of the Rickettsial Zoonoses Branch, Division of Vector-Borne Diseases, National Center for Emerging and Zoonotic Infectious Diseases, Centers for Control Disease and Prevention, for diagnostic testing to confirm results by using >1 of the following assays: PanR8 *Rickettsia* spp. real-time PCR (*4*), RCKr *Rickettsia* spp. real-time reverse transcription PCR (*6*), and RT27 and RT12 for *R. typhi* (D. Chinchilla, unpub. data). We attempted sequencing for all specimens that had positive results by using the 17kDa (TG and SFG) (*7**,**8*) and *ompA* (SFG) (*9**,**10*) gene targets.

We detected *Rickettsia* spp. nucleic acid in 8 samples (7.7%). Of those, 2 (1 whole blood and 1 serum) were identified as *R. typhi* (GenBank accession no LS992663.1) by sequencing of the 17-kDa gene targeting TG *Rickettsia*. The remaining 6 samples that were positive for *Rickettsia* spp. could not be further identified by sequencing, probably because of low copy numbers (Appendix). The samples corresponding to the 2 murine typhus cases were submitted in March and October 2019.

Both murine typhus patients required hospitalization and had severe disease develop. The patients lived in a rural area in Cartago, a province located in the Central Valley of Costa Rica (Figure). Both patients had fever and moderate symptoms at the early stages but later progressed to severe disease (Table). One of the patients required admission to the intensive care unit and mechanical ventilation, and the other patient died of superimposed nosocomial pneumonia (Table). Neither patient had a rash, which occurs in up to 50% of the patients with murine typhus and is often considered suggestive of rickettsial disease. At hospital admission, both patients had fever of unknown origin, but rickettsiosis was not considered in the clinical diagnosis because of the lack of knowledge of the incidence of rickettsial disease in Costa Rica and the consideration that a rash is expected to occur.

**Figure:**
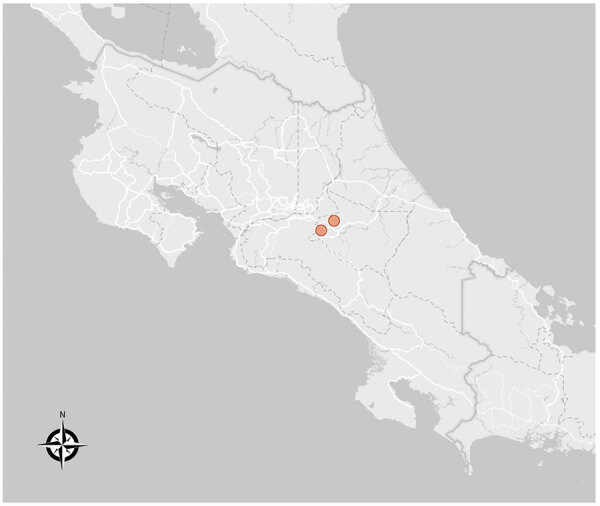
Location of 2 cases of murine typhus (colored circles), Costa Rica.

**Table:** Characteristics of 2 patients confirmed to have murine typhus, Costa Rica, 2019

Characteristic	Patient 1	Patient 2
Age, y/sex	30/M	46/F
Presumptive diagnosis upon arrival	Leptospirosis, ehrlichiosis	Lyme disease, brucellosis, dengue fever
Travel history	Visited Cocles Beach on the Atlantic coast of Costa Rica	Not indicated
Days after onset of symptoms	Sample drawn within 7 d	Sample drawn within 9 d
Early symptoms	Fever, joint pain, myalgia, chills, headache, abdominal pain, dehydration, diarrhea, cough	Fever, chills, headache, vomiting, cough
Late symptoms	Jaundice, gum bleeding, multiple organ failure	Sepsis
Hospital ward	Admitted to intensive care unit, required mechanical ventilation	Admitted to infectious disease ward
Outcome	Recovered after 15 d treatment with doxycycline and tigecycline	Died from nosocomial pneumonia, septic shock, and renal failure (as stated in certificate of death)
Seroconversion in convalescent-phase sample*	No convalescent-phase sample	Titer of IgG against *Rickettsia typhi* 1:64

*By immunofluorescence for IgG against spotted fever group and typhus group *Rickettsia* (Fuller Laboratories, http://www.fullerlabs.com).

We evaluated samples by using the PanR8 assay as part of the laboratory differential diagnosis assays performed for samples suspected of indicating a tickborne disease. Patient 1 was initially suspected of having leptospirosis or ehrlichiosis, and patient 2 was suspected of having Lyme disease, brucellosis, or dengue; all of those conditions are febrile diseases that have similar symptoms at early stages. Subsequent sequencing using 17-kDa TG primers identified *R. typhi* DNA within each sample. Patient 2 showed seroconversion to TG in a convalescent-phase sample (titer 1:64)

In conclusion, we report 2 cases of murine typhus in Costa Rica, confirm the presence of *R. typhi* by sequencing, and document the occurrence of severe disease. Our findings indicate that *R. typhi* is actively circulating in Costa Rica and is capable of causing severe disease. Early clinical and laboratory diagnosis of SFG and TG rickettsiosis in Costa Rica will ensure timely treatment to prevent complications and deaths. Laboratory confirmation of the specific rickettsial agent is also critical to support epidemiologic interventions, including vector control and community and physician education.

Appendix Additional information on severe *Rickettsia typhi* infections, Costa Rica.
