# Supplementary material for: Severe Rickettsia typhi Infections, Costa Rica
Source: Emerg Infect Dis. 2023 Nov;29(11):2374–6. doi: 10.3201/eid2911.221561 (PMC10617344; doi:10.3201/eid2911.221561)
Supplement: Appendix — Additional information on severe Rickettsia typhi infections, Costa Rica. [file 22-1561-Techapp-s1.pdf]

Article DOI: https://doi.org/10.3201/edi2911.221561

*EID cannot ensure accessibility for Supplemental Materials supplied by authors. Readers who have difficulty accessing supplementary content should contact the authors for assistance.*

# Severe *Rickettsia typhi* Infections, Costa Rica

## Appendix

**Appendix Table.** Test results for *Rickettsia* sp.–positive samples, Rickettsial Zoonoses Branch Laboratory, CDC

| Sample | Sample type | PanR8* | SFG OmpA† | SFG 17-kDa‡ | TG 17-kDa§ | RCk-r RT-PCR¶ | RT27 RT12# | Sequencing |
|---|---|---|---|---|---|---|---|---|
| 014 | Whole blood | Positive | Positive | ND** | Positive | Positive | NT | Undetermined |
| 051 | Serum | Positive | ND | ND | Positive | Positive | NT | Undetermined |
| 056 | Serum | Positive | Positive | ND | Positive | ND | NT | Undetermined |
| 094 | Swab | ND | NT†† | NT | NT | Positive | NT | NT |
| 147 | Biopsy | ND | NT | NT | NT | Positive | NT | NT |
| 159‡‡ | Whole blood | Positive | ND | ND | Positive | Positive | Positive | *R. typhi* |
| 165 | Serum | ND | NT | NT | NT | Positive | NT | NT |
| 201§§ | Serum | ND | ND | ND | Positive | Positive | Negative | *R. typhi* |

*PanR8 *Rickettsia* spp. real-time PCR.
†Spotted Fever Group *OmpA* real-time PCR.
‡17 Spotted Fever Group 17-kDa antigen real-time PCR.
§Typhus Group 17-kDa antigen real-time PCR.
¶RCKr *Rickettsia* spp. real-time reverse transcription real-time PCR.
#*Rickettsia typhi* 27RT and *Rickettsia typhi* 12RT real-time PCR.
**ND not detected.
††NT not tested.
‡‡Sample from Patient 1.
§§Sample from Patient 2.
